# GNSS NLOS Signal Classification Based on Machine Learning and Pseudorange Residual Check

**DOI:** 10.3389/frobt.2022.868608

**Published:** 2022-05-05

**Authors:** Tomohiro Ozeki, Nobuaki Kubo

**Affiliations:** Department of Maritime Systems Engineering, Tokyo University of Marine Science and Technology, Tokyo, Japan

**Keywords:** GNSS, multipath, support vector machine, DGNSS, NLOS, pseudorange residual

## Abstract

Global navigation satellite system (GNSS) positioning has recently garnered attention for autonomous driving, machine control, and construction sites. With the development of low-cost multi-GNSS receivers and the advent of new types of GNSS, such as Japan’s Quasi-Zenith Satellite System, the potential of GNSS positioning has increased. New types of GNSS directly increase the number of line-of-sight (LOS) signals in dense urban areas and improve positioning accuracy. However, GNSS receivers can observe both LOS and non-line-of-sight (NLOS) signals in dense urban areas, and more NLOS signals are observed under static conditions than under dynamic conditions. The classification of LOS and NLOS signals is important, and various methods have been proposed, such as C/N0, using three-dimensional maps, fish-eye view, and GNSS/inertial navigation system integration. Multipath detection based on machine learning has also been reported in recent years. In this study, we propose a method for detecting NLOS signals using a support vector machine (SVM) classifier modeled with unique features that are calculated by receiver independent exchange format-based information and GNSS pseudorange residual check. We found that using both the SVM classifier and GNSS pseudorange residual check effectively reduced the error due to NLOS signals. Several static tests were conducted near high-rise buildings that are likely to receive some NLOS signals in downtown Tokyo. For all static tests, the percentage of positioning errors within 10 m in the horizontal positioning error was improved by >80% by detecting and eliminating satellites receiving NLOS signals.

## 1 Introduction

Global navigation satellite system (GNSS) positioning has recently been used in autonomous driving, machine control, and at construction sites. The development of new low-cost multi-GNSS receivers and the advent of new types of GNSS, such as the Quasi-Zenith Satellite System (QZSS) in Japan, have increased the potential of GNSS positioning. One of the greatest advantages of GNSS positioning is the ease with which absolute positions may be obtained, such as in the Earth-centered, Earth-fixed coordinate system (ECEF). Heights can be obtained from barometers and precision maps, but there are limited ways to easily obtain horizontal positions, which is another advantage conferred by GNSS positioning. Additionally, the advent of new types of GNSS has increased the number of line-of-sight (LOS) signals and improved positioning accuracy in challenging locations, such as dense urban areas. However, GNSS receivers observe both LOS signals and non-line-of-sight (NLOS) signals in these areas. NLOS signals have large multipath errors, which is the reason for the degrading positioning accuracy in dense urban areas (Kubo et al., 2020). Under kinematic conditions, the environment around the GNSS antenna is dramatically changed and the GNSS receiver is less likely to track NLOS signals, but under static conditions, the environment around the GNSS antenna is unchanged and more NLOS signals can be tracked by the GNSS receiver (Kubo et al. (2017). This can be a significant challenge, for example, in the case of landslide monitoring and parking locations.

Classifying LOS and NLOS signals correctly is essential for improving GNSS positioning accuracy, and various methods of achieving such improvements have been proposed, such as C/N0, the use of three-dimensional (3D) maps, or fish-eye view. Kubo et al. (2020) proposed a method for classifying NLOS signals using continuous C/N0 time series, where NLOS signals were detected by analyzing the C/N0 time series and GNSS pseudorange residual check calculated by the least-squares method, through which the differential GNSS (DGNSS) positioning result was dramatically improved. Classification methods using machine learning have also been proposed recently. Suzuki and Amano (2021) proposed that the correlation output of a GNSS signal can be classified using machine learning. They used the shape of a multi-correlator for features, and 97.7% of the NLOS signals were correctly discriminated. However, it is difficult to obtain the shape of a multi-correlator when using a commercial GNSS receiver. Xu et al. (2018) proposed a classification method based on machine learning and using receiver independent exchange format (RINEX)-based information. They compared various machine learning algorithms including k-nearest neighbors, neural network, support vector machine (SVM), and decision tree, and SVM was most effective for classifying LOS and NLOS signals. Xu et al. (2020) proposed a classification method based on an SVM using RINEX and estimated positions using GNSS shadow matching after classification, and the mean error in the cross-street direction was decreased from 10.27 to 1.44 m. Despite this, 3D maps are not suitable for commercial use because of their uncertain availability and computational cost. For large-scale commercial use, a positioning method that can be handled by a single software program, with as little additional equipment and cost as possible, is preferred. The goal of this study was to detect NLOS signals using an SVM classifier modeled with unique features and, thus, to improve horizontal DGNSS positioning results in an urban area.

## 2 Materials and Methods

### 2.1 SVM Classifier

An SVM is a supervised learning tool that generates input–output mapping functions from a set of labeled training data (Wang, 2005). In this study, the SVM classifier outputted LOS and NLOS signals and defined LOS signals as negative and NLOS signals as positive. We implemented an SVM classifier using Scikit-learn (Pedregosa et al., 2011), an open-source machine learning library for the Python programming language. The SVM classifier implemented by Scikit-learn requires tuning of the parameters called “hyper-parameters.” We chose a radial basis function kernel (RBF), which is generally used for nonlinear classification, while the other parameters were selected using a grid search (Min and Lee, 2005).

Xu et al. (2020) previously used four features—the signal-to-noise ratio (SNR), elevation angle (EA), normalized pseudorange residual (NPR), and pseudorange rate consistency (PRC). As for the SNR and EA, it is well known that both are closely related to the NLOS signal (Tokura and Kubo et al., 2017). SNR can be obtained by RINEX, and EA can be calculated by the satellite position estimated by the ephemeris and the approximate user position.

The pseudorange residual is valid for use with machine learning (Hsu, 2017). It is calculated by the least-squares method and usually becomes large for NLOS signals. However, the positioning result from the least-squares method has large errors under multipath environments and the pseudorange residual does not perfectly indicate the difference between LOS and NLOS signals. For this reason, the pseudorange residual was normalized at each epoch. The NPR can be expressed as

$$NPR_{s}=\frac{Pr_{s}-Pr_{min}}{Pr_{max}-Pr_{min}},$$

where $Pr_{max}$ and $Pr_{min}$ were calculated by the maximum and minimum pseudorange residuals at each epoch, respectively, and $Pr_{s}$ is the pseudorange residual of satellite s .

PRC is defined as the difference in the changing rate of pseudorange from the pseudorange and Doppler shift (Hsu, L. (2017). The changing rate of pseudorange from the pseudorange can be expressed as

$$\Delta P=P\left(t_{i}\right)-P\left(t_{i-1}\right),$$

where $P\left(t_{i}\right)$ is the pseudorange in the i th epoch. The changing rate of the pseudorange can also be calculated by the Doppler shift as

$$\Delta \rho =\left(-\lambda_{s}f_{s}\right)\text{Δ}t,$$

where $\lambda_{s}$ is the wavelength of satellite s , $f_{s}$ is the Doppler shift of satellite s in unit of Hz, and Δt is the time difference of $t_{i}$ and $t_{i-1}$ . PRC can be expressed as

PRC=ΔP−Δρ

The pseudorange can be estimated by the receiver-code tracking loop, and the Doppler shift can be estimated by the frequency tracking loop in the GNSS receiver. For an LOS signal, the PRC is 0, but for an NLOS signal, the PRC is not always 0 because the receiver-code tracking loop is more affected by multipaths than by the frequency tracking loop.

**FIGURE1:**
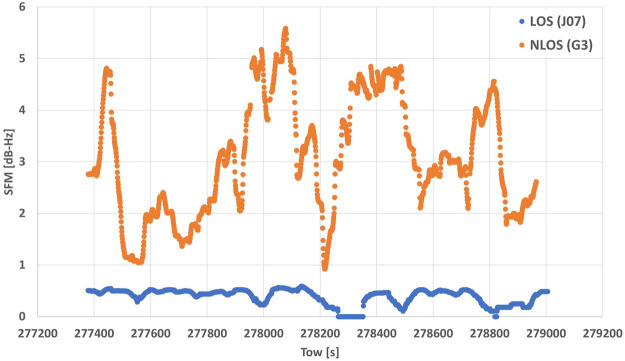
LOS and NLOS time series of the magnitude of fluctuation in the SFM.

Previous studies have shown that there are more methods other than just these four to characterize NLOS signals. Tokura and Kubo (2017) proposed an SNR-based satellite selection method that uses the magnitude of variability in the SNR (hereafter, the “SNR fluctuation magnitude” or SFM). Because NLOS signals exhibit large fluctuations in their SNR time series, the SFM is an indicator of the magnitude of such fluctuations. Tokura and Kubo (2017) calculated the SFM using the moving standard deviation of the difference between the observed and estimated SNRs, which sets the elevation-dependent threshold of the SNR (Suzuki et al., 2004; Kubo et al., 2005; Shirai & Kubo, 2012). The disadvantage of this approach is that it is difficult to set this threshold. Instead, we calculated the SFM in this study by moving the standard deviation of the SNR, expressed as

$$SFM\left(i\right)=\sqrt{\frac{1}{N}\overset{i}{\underset{n=i-N}{\sum }}{\left(SNR\left(t_{i-n}\right)-SNR_{mean}\right)}^{2}},$$

where *N* is the window size and $SNR\left(t_{i-n}\right)$ is the SNR in the i−n th epoch and $SNR_{mean}$ is the mean of the SNR. Figure 1 shows a comparison of the LOS and NLOS SFM time series in a multipath environment. The window size was set to 120 epochs. The LOS of the mean residual was 1.56 m, but that of the NLOS was 55.90 m. These results indicate that SFM can be a good indicator of LOS versus NLOS signals.

We generated a classifier with five features—SNR, EA, NPR, PRC, and SFM. For the SFM, the window size for calculating the moving standard deviation influenced the accuracy of the SVM classifier. In this study, we set various window sizes (30, 60, 90, 120, 180, and 240 epochs) and considered the best window size for the accuracy of the SVM classifier. The SVM classifier was then used to classify all signals that could be used for positioning at each epoch.

### 2.2 Datasets

**FIGURE 2:**
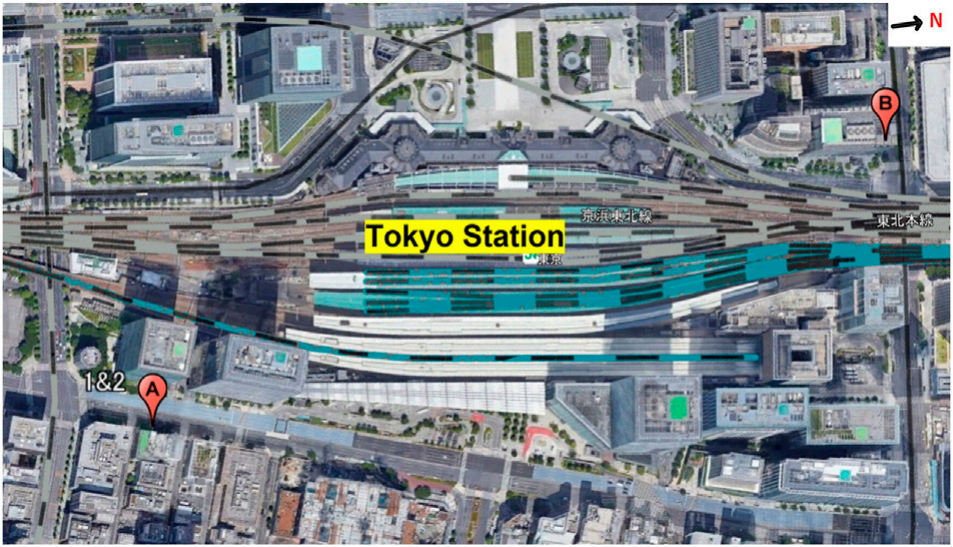
Map showing locations A and B relative to Tokyo Station.

Three datasets were used in this study; Figure 2 shows the data collection locations. The area around Tokyo Station is surrounded by skyscrapers over 100 m in height, and the DGNSS positioning error easily reached 100 m. Locations A and B are marked in Figure 2. For all datasets, a U-blox F9P and a standard patch antenna (ANN-MB-00–00) installed on the roof of a parked car (U-blox, Switzerland) were used. In the reference station, a U-blox F9P was used and the GNSS antenna was a Trimble Zephyr 2 Geodetic (Trimble, Inc., United States). All datasets were recorded at 1 Hz.

**FIGURE 3:**
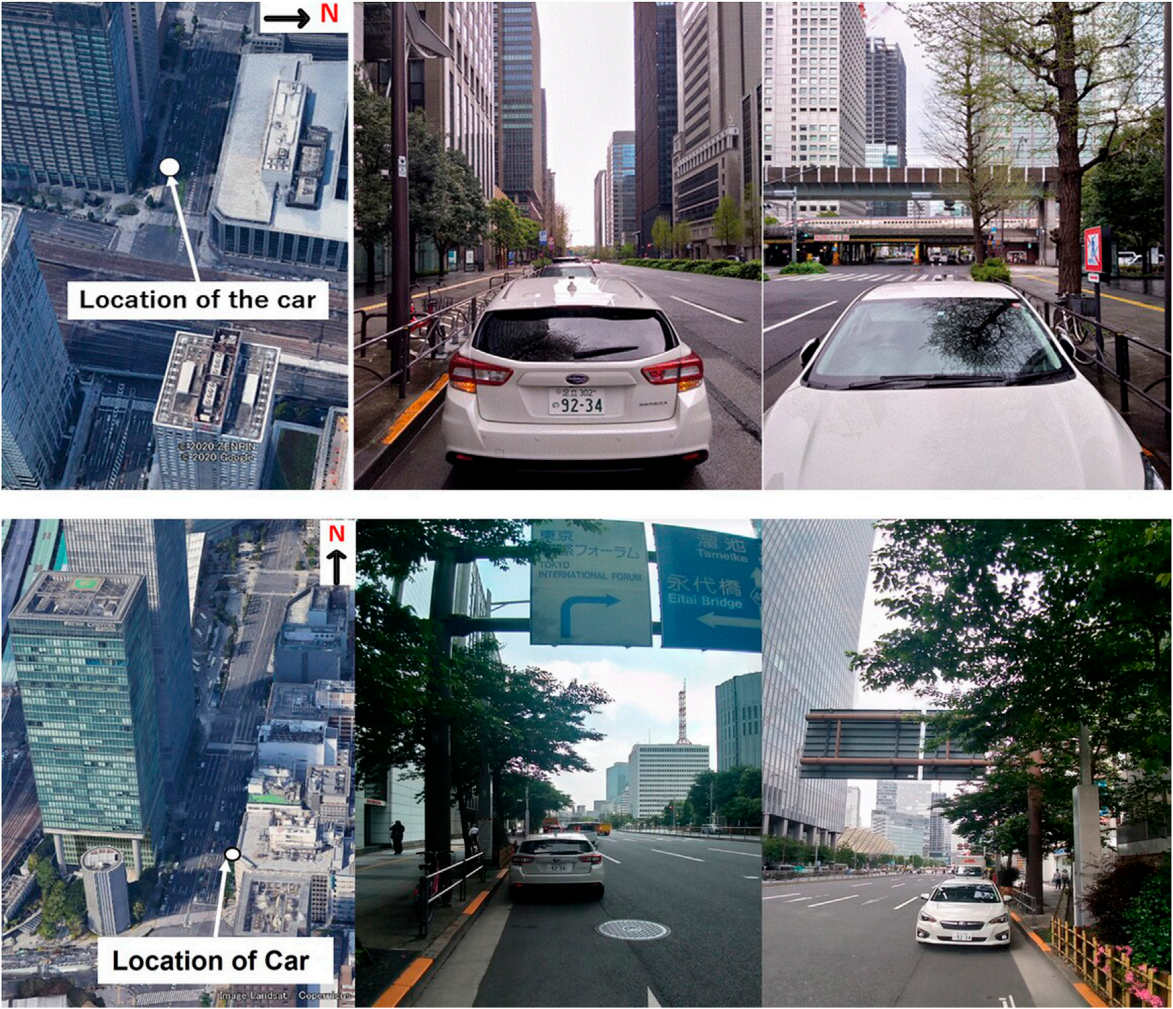
Environment of location A (upper) and environment of location B (lower). The white circle represents the location of the GNSS antenna.

The upper part of Figure 3 shows detailed images around location A and the antenna. As can be seen, it was likely that NLOS signals would be received from the higher buildings at the azimuth of 290°. There were several high-rise buildings of different heights and several trees on both sides of the antenna. The lower part of Figure 3 shows detailed images around location B and the antenna. As in location A, it was also likely at this location that NLOS signals would be received from the higher buildings at the azimuth of 180°, and there were several high-rise buildings of different heights on both sides of the antenna.

Datasets (1) and (2) were collected at location A but were 50 cm apart when comparing the precise position. Dataset (3) was collected at location B. All datasets were accompanied by reference station data obtained at the Tokyo University of Marine Science and Technology Etchujima Campus located ∼3 km from Tokyo Station. Table 1 shows the dates and times when the datasets were collected. As for the reference position, we adopted an RTK-GNSS Fix solution outputted by U-blox F9P, and we confirmed that this position was correct to an accuracy of approximately 5 cm in post-processing by positioning software developed by our laboratory.

**TABLE 1:** Details of each dataset.

Datasets	Location	Date and time (GPS tow [s])	Train or test
(1)	Location A	03/04/2020, 461880–463500	Train
(2)	Location A	27/05/2020, 540181–541800	Test
(3)	Location B	18/04/2020, 277379–279005	Test

Dataset (1) and reference station data were used for training the SVM classifier, and datasets (2) and (3) were used for testing the SVM classifier. For datasets (1) and (2), the locations of data collection were almost the same, but the collection times differed. The locations and times of data collection were different for datasets (1) and (3). Therefore, these datasets were suitable for evaluating the SVM classifier.

### 2.3 Labels

In the testing and evaluation of our SVM classification, we estimated accurate pseudorange residuals and determined the labels for classification. Kubo et al. (2020) proposed an estimation of the precise receiver clock error and pseudorange residuals. Pseudorange measurements include errors from the receiver clock, satellite clock, ionosphere, troposphere, and multipath + noise. In pseudorange positioning, the multipath + noise error is the residual of the pseudorange

$$P=\rho +c\left(dt_{\mathrm{rcv}}-dT_{\mathrm{sat}}\right)+\mathrm{ion}+\mathrm{tropo}+\mathrm{mp}+\varepsilon ,$$

where *P* is the pseudorange (m), ρ is the geometrical range (m), *c* is the speed of light (m/s), $\;dt_{rcv}$ is the receiver clock error (m), $\;dT_{sat}$ is the satellite clock error (m), *ion* is the ionospheric error (m), *tropo* is the tropospheric error (m), *mp* is the multipath error (m), and  ε is the noise error (m).

In this study, the predicted geometrical range was set as the distance between the reference position of the antenna and the satellite position estimated by ephemeris. The satellite clock, ionospheric, and tropospheric errors are considered to be in part eliminated by the DGNSS correction data, which were calculated at the base station. Additionally, the clock bias of GPS and other satellite systems was eliminated by the DGNSS correction data. Therefore, a receiver clock was needed to estimate the multipath + noise. If the receiver clock was estimated within a few meters, then the multipath + noise can be estimated with the same accuracy. Usually, the receiver clock error was estimated by pseudorange positioning and estimation accuracy if the receiver clock errors were within several meters under open sky conditions. However, in multipath environments, the estimation accuracy of receiver clock errors deviated over tens of meters because of multipath errors. In this study, we tracked a strong signal from the Japanese QZSS even near high-rise buildings in Tokyo as at least one QZSS remained at a very high EA > 80°. In practice, the accuracy of the pseudorange of the highest satellite is within 1.0 m. Using the receiver clock error estimated with the pseudorange of the highest EA and reference positions of the antenna, the multipath + noise could be expected within 1.0 m. The NLOS signal multipath errors usually exceed 10 m. Here, if the multipath errors exceeded 10 m, they represented an NLOS signal.

### 2.4 Analytical Strategy

Multipath errors caused by NLOS signals generally have a greater impact on the pseudorange than the carrier phase, and the accuracy of carrier-phase positioning, such as real-time kinematic, is affected by the pseudorange positioning. In this study, we adopted DGNSS positioning as it is suitable for evaluating positioning errors because it is less affected by tropospheric, ionospheric, and satellite clock errors. Table 2 shows the common parameters of our analysis.

**TABLE 2:** Common analytical parameters.

Item	Parameter
GNSS receiver	U-blox F9P (rover/base)
GNSS antenna	ANN-MB-00-00 (rover)/Trimble Zephyr 2 Geodetic (base)
Mask angle	15°
Minimum SNR	30 dB-Hz
Threshold for residuals	10 m
Satellites	GPS/QZSS/GALILEO/BDS/GLONASS
Interval	1 Hz
Minimum satellites	5

**FIGURE 4:**
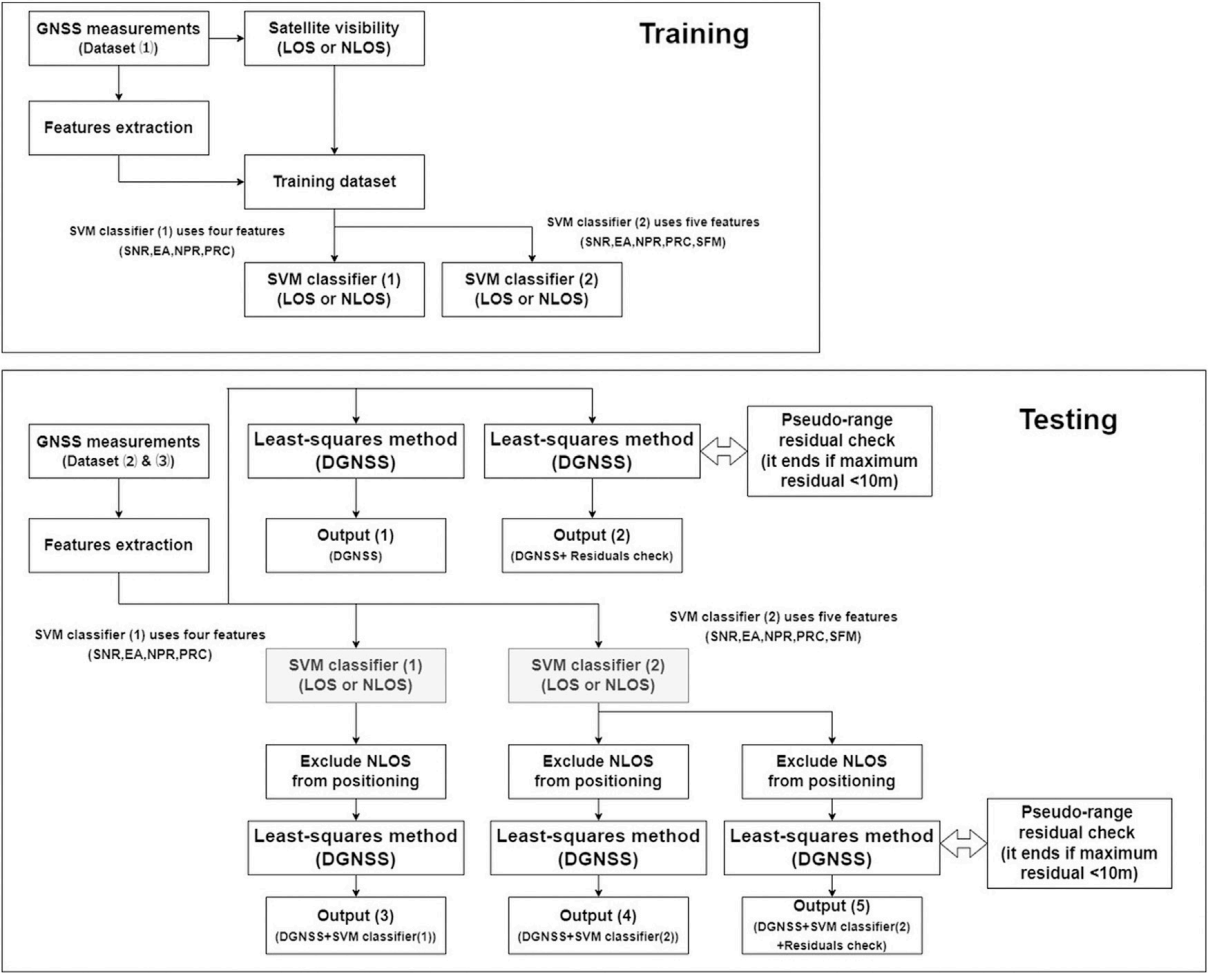
Workflow of training (upper) and testing (lower).

Figure 4 shows the workflow of this study. We extracted five features (SNR, EA, NPR, PRC, and SFM) and determined labels for classification during training. To validate the SFM, we generated two SVM classifiers—SVM classifier (1), which used four features (SNR, EA, NPR, and PRC), and SVM classifier (2), which used five features (SNR, EA, NPR, PRC, and SFM). During the testing phase, signals classified as NLOS were excluded by DGNSS positioning. There were five outputs.

・Output (1) was the DGNSS position from satellites that could be observed and used to assess the effect of pseudorange residual check.
・Output (2) was the DGNSS position from satellites that were not excluded by the pseudorange residual check.
・Output (3) was the DGNSS positioning using SVM classifier (1).
・Output (4) was DGNSS positioning using SVM classifier (2).
・Output (5) was the DGNSS positioning using SVM classifier (2) and pseudorange residual check.

For the SFM, we set various window sizes (30, 60, 90, 120, 180, and 240 epochs) and considered the best window size from the results of the SVM classifier accuracy across all datasets.

The positioning software developed by our laboratory was used. As for the pseudorange residual check, the position and pseudorange residuals were first calculated by the least-squares method using all satellites. If the maximum pseudorange residual exceeded the threshold, the satellite with the maximum pseudorange residual was excluded. This process continued until the maximum pseudorange residual fell below the threshold or the number of satellites was insufficient (Jiang et al., 2011).

## 3 Results

### 3.1 SVM Classifier

The parameters of the SVM classifier, the regularization parameter, and the kernel coefficient were set equal to 10, and the scales were selected via grid search (Min & Lee, 2005). There are four types of statistics used to evaluate binary classifications—true positive (TP), true negative (TN), false positive (FP), and false negative (FN) (Sokolova & Lapalme, 2009). In this study, accuracy was chosen as the metric for evaluating the SVM classifier. Accuracy can be expressed as

$$\mathrm{Accuracy}=\frac{\mathrm{TP}+\mathrm{TN}}{\mathrm{TP}+\mathrm{TN}+\mathrm{FP}+\mathrm{FN}}$$

Table 3 shows the accuracy of the SVM classifier. Using the SFM, the accuracy of the classifier increased by >15% with the testing. Regarding the best SFM window size, there was no significant difference in the accuracy when the window size was 60 epochs or more. Therefore, we selected 120 epochs, which maximized the accuracy in dataset (2).

**TABLE 3:** Accuracy of the SVM classifier.

Accuracy (%)	Dataset (1)	Dataset (2)	Dataset (3)
SVM classifier (1)	87.61	64.50	76.17
SVM classifier (2)_SFM (30 epochs)	95.38	83.98	91.42
SVM classifier (2)_SFM (60 epochs)	95.90	86.35	90.83
SVM classifier (2)_SFM (90 epochs)	96.18	87.72	90.80
SVM classifier (2)_SFM (120 epochs)	96.23	88.00	91.52
SVM classifier (2)_ SFM (180 epochs)	95.76	87.78	92.54
SVM classifier (2)_ SFM (240 epochs)	95.63	87.59	92.44

### 3.2 Positioning Results

We evaluated the horizontal DGNSS error using six statistics: mean error, maximum error, standard deviation (SD), and the percentages of positioning errors within 3 m, 10 m, and 30 m.

#### 3.2.1 Dataset (2)

**FIGURE 5:**
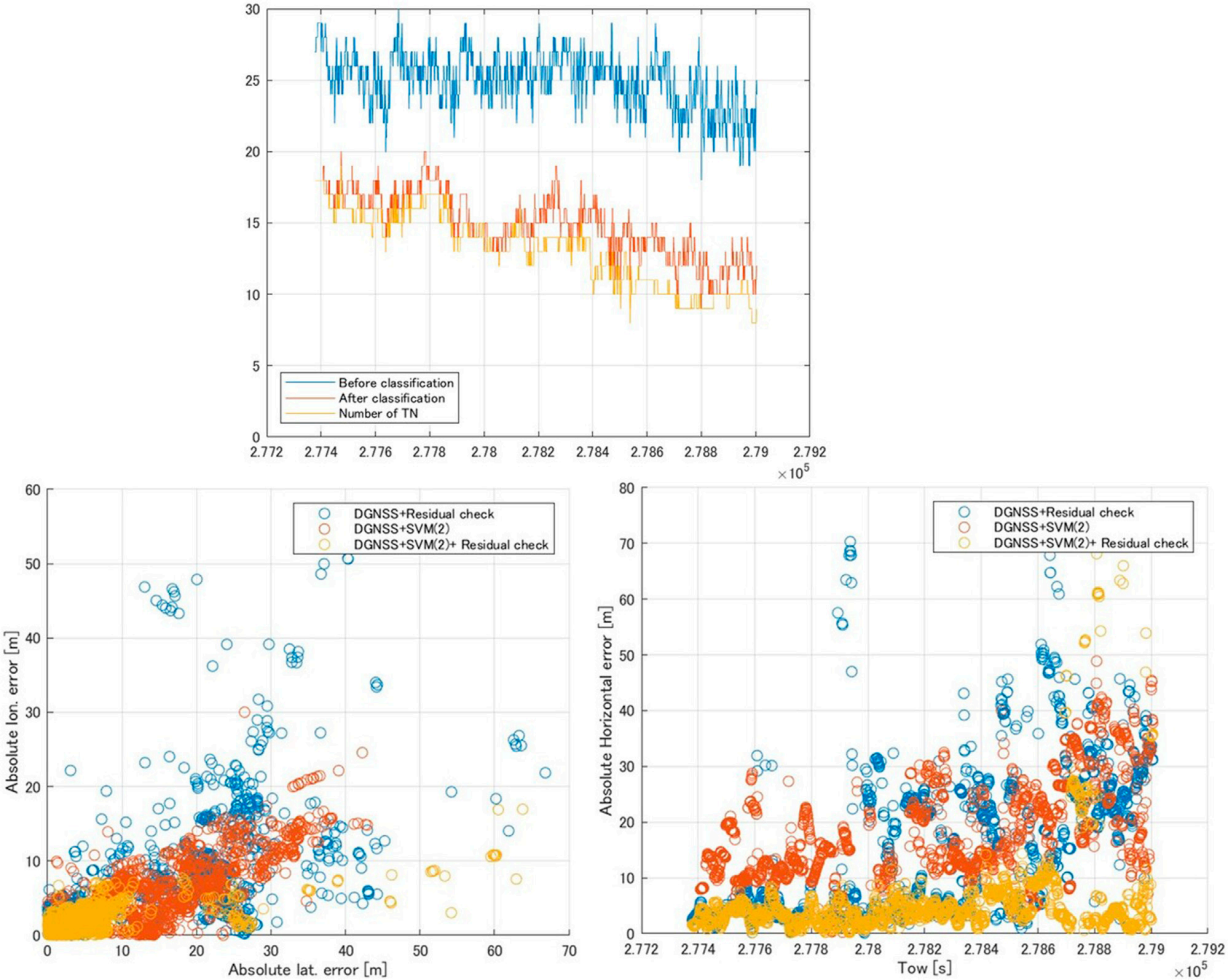
Time series of the number of satellites using dataset (2) (upper) and comparison of the DGNSS positioning error in dataset (2) (lower). The “after classification” period represents the number of satellites determined to be LOS by SVM classifier (2), and the “number of TN” is the number of true negative (TN) satellites (LOS signals) at each epoch.

The upper part of Figure 5 shows the time series of the number of satellites used in dataset (2). The mean number of satellites before and after classification was 25 and 14.9, respectively, while the number of TN satellites was 13.1. Therefore, SVM classifier (2) detected approximately 10 NLOS signals in each epoch. Additionally, the mean numbers of TPs, FPs, and FNs in each epoch were 9.0, 1.1, and 1.8 satellites, respectively.

Table 4 shows a comparison of the horizontal positioning errors in dataset (2). Comparing DGNSS + SVM classifier (1) and DGNSS + SVM classifier (2), the percentage of positioning errors within 10 m was especially improved because the accuracy of SVM classifier (2) was 25% higher than that of SVM classifier (1) (see also Table 3). However, it did not exceed the values of the DGNSS + residual check. For the combination of DGNSS + SVM classifier (2) + residual check, the percentage of positioning errors within 10 m was dramatically improved compared with other results, while the maximum error was worse due to the fact that FNs in SVM classifier (2) and pseudorange residual check were incorrect. To make it easier to visually understand the improvement of the horizontal positioning results, the lower part of Figure 5 shows the DGNSS positioning errors with the DGNSS + residual check, DGNSS + SVM classifier (2), and DGNSS + SVM classifier (2) + residual check. The DGNSS + SVM classifier 2) + residual check yielded the smallest error.

**TABLE 4:** Comparison of the horizontal positioning error of dataset (2).

Dataset (2) (1,625 epochs)	DGNSS output (1)	DGNSS + residual check output (2)	DGNSS + SVM classifier (1) output (3)	DGNSS + SVM classifier (2) output (4)	DGNSS + SVM classifier (2) + residual check output (5)
Mean error	27.68 m	14.12 m	29.69 m	15.99 m	5.77 m
Maximum error	93.14 m	70.32 m	68.24 m	48.89 m	68.16 m
SD	10.39m	13.10 m	9.42 m	9.70 m	7.82 m
Percentage (<3 m)	1.05%	17.17%	0.00%	8.12%	33.97%
Percentage (<10 m)	5.78%	55.14%	0.49%	27.51%	91.63%
Percentage (<30 m)	59.69%	88.00%	57.97%	89.66%	98.22%

#### 3.2.2 Dataset (3)

**FIGURE 6:**
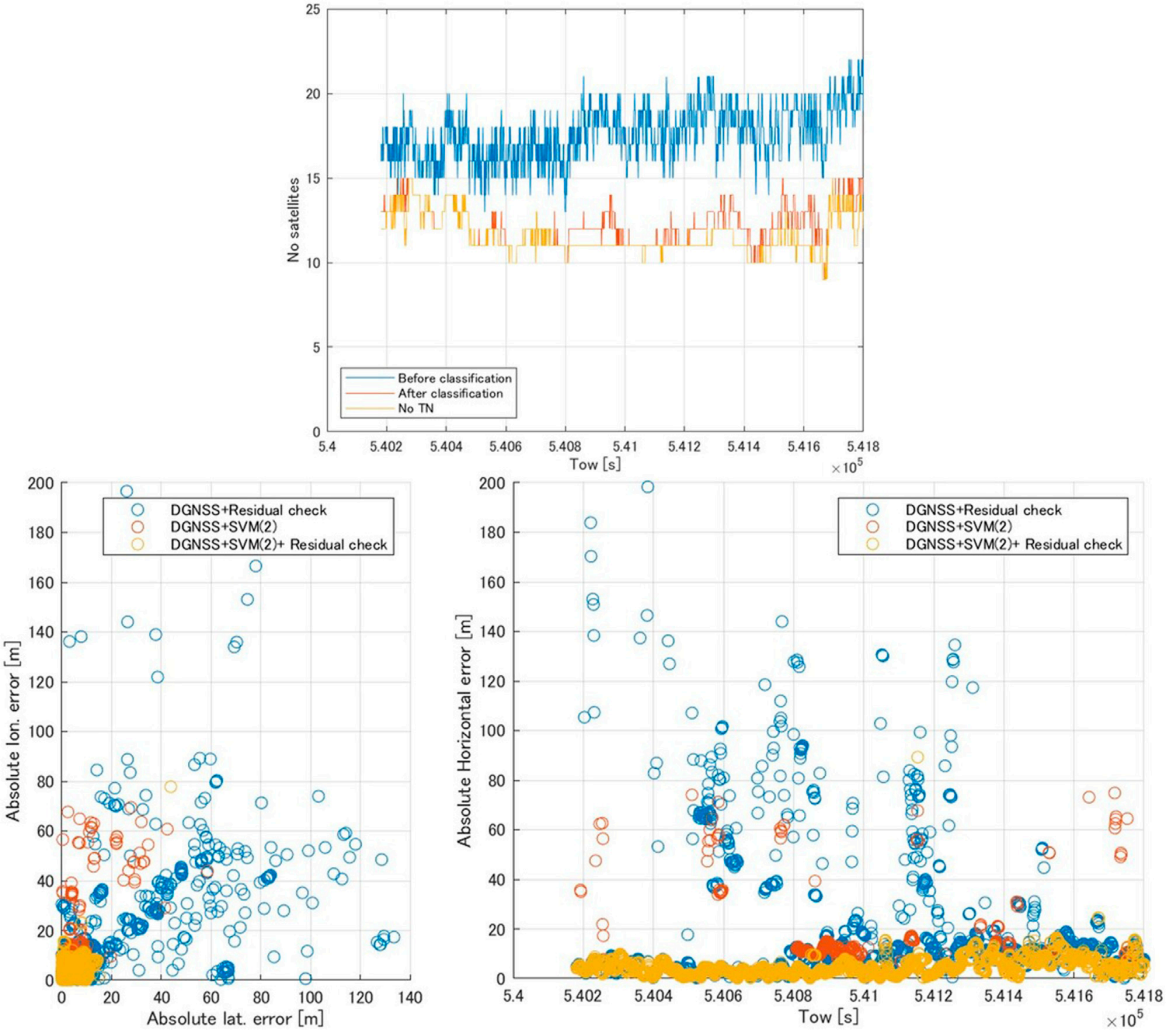
Time series of the number of satellites using dataset (3) (upper) and comparison of DGNSS positioning error in dataset (3) (lower). The “after classification” period represents the number of satellites determined to be LOS by SVM classifier (2), and the “number of TN” is the number of true negative (TN) satellites (LOS signals) at each epoch.

The upper part of Figure 6 shows the time series of the number of satellites used in dataset (3). The mean number of satellites before and after classification was 17.6 and 12.2, respectively, and the number of TN satellites was 11.6. Therefore, SVM classifier (2) detected approximately five NLOS signals in each epoch. Moreover, the mean numbers of TPs, FPs, and FNs in each epoch were 4.6, 0.6, and 0.8 satellites, respectively.

Table 5 shows a comparison of the horizontal positioning errors in dataset (3), and the lower part of Figure 6 shows the DGNSS positioning error with the DGNSS + residual check, DGNSS + SVM classifier (2), and DGNSS + SVM classifier (2) + residual check. Except for the maximum error, all statistics showed improvements as great as those in dataset (2). In the case of the DGNSS + SVM classifier (2) + residual check, the percentage of positioning errors within 10 m was improved by >80% compared to the DGNSS alone, and >90% of all horizontal positioning errors were within 10 m.

**TABLE 5:** Comparison of the horizontal positioning error of dataset (3).

Dataset (3) (1,620 epochs)	DGNSS output (1)	DGNSS + residual check output (2)	DGNSS + SVM classifier (1) output (3)	DGNSS + SVM classifier (2) output (4)	DGNSS + SVM classifier (2) + residual check output (5)
Mean error	51.43 m	18.15 m	17.46 m	7.52 m	4.93 m
Maximum error	263.66 m	198.33 m	121.48 m	74.85 m	89.28 m
SD	33.68 m	26.19 m	14.88 m	9.44 m	3.94 m
Percentage (<3 m)	1.49%	14.44%	7.96%	29.07%	34.57%
Percentage (<10 m)	7.59%	54.32%	34.14%	78.89%	92.35%
Percentage (<30 m)	33.15%	83.89%	87.41%	96.72%	99.94%

### 3.3 Kinematic Test and Results

**FIGURE 7:**
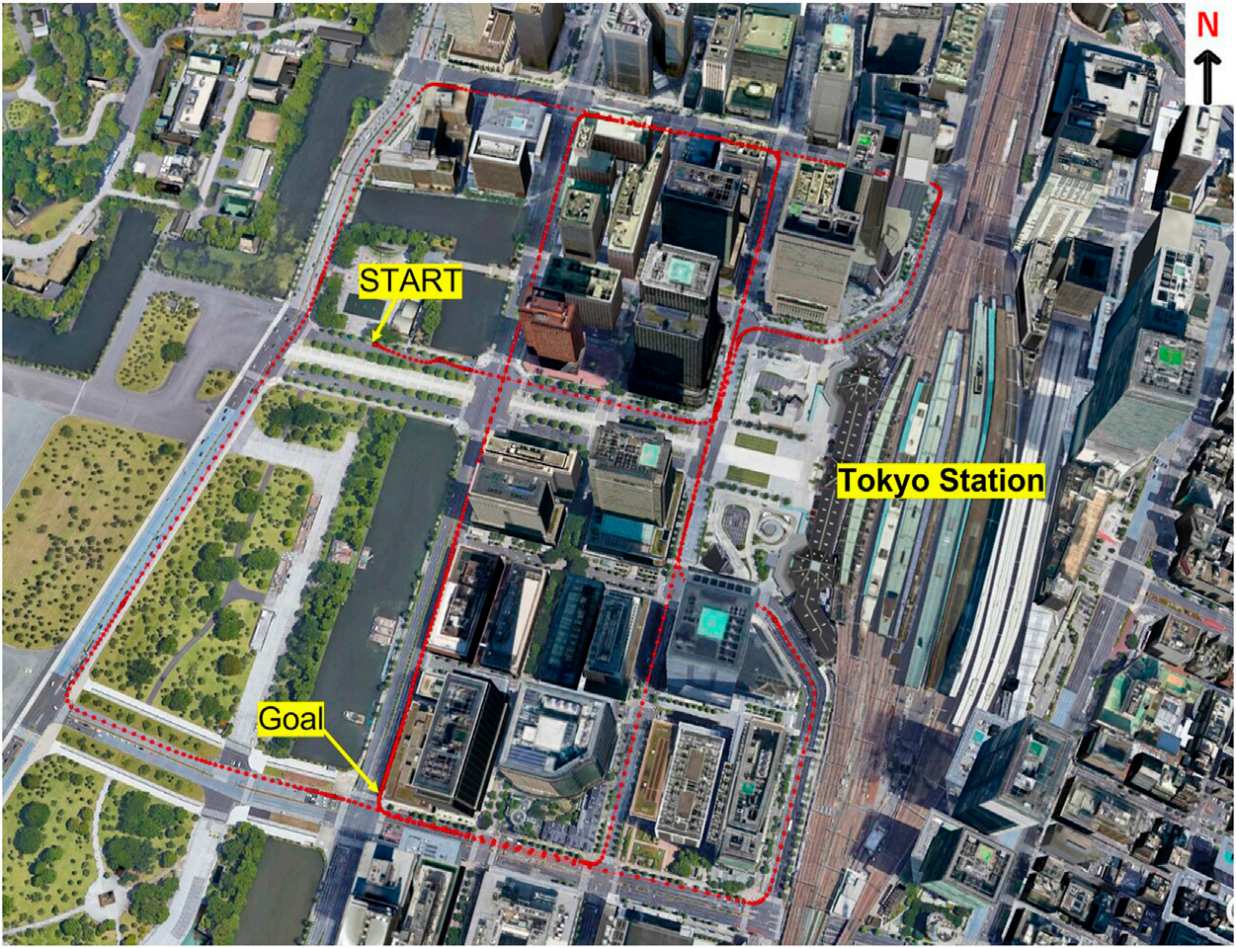
Environment of the route in the kinematic test.

To find the limitations of the proposed method, we conducted a kinematic test. The GNSS antenna was installed in a car that traveled along the route shown in Figure 7. We collected data and analyzed the DGNSS positioning. The route included skyscrapers around Tokyo Station in Tokyo, Japan, and the DGNSS positioning error easily reached several tens of meters or more in this area. The total data recording period was 1,811 s, and the data were recorded at 1 Hz. GPS-703-GGG-HV (Novatel, Inc., Canada), which is a survey-grade GNSS antenna, was used for this experiment. The reference position was obtained using POS LVX (Applanix, Inc, Canada). The other settings were the same as in Table 2.

Table 6 shows the accuracy of the SVM classifier in the kinematic test and dataset (1). Compared to the experiment under the static conditions, there was no contribution of SFM to the accuracy. This is because the positioning environment changes from time to time under the kinematic conditions, and the signal frequently changes from LOS to NLOS or NLOS to LOS. Hence, we used SVM classifier (1) in the kinematic test.

**TABLE 6:** Accuracy of the SVM classifier in the kinematic test.

Accuracy (%)	Dataset (1)	Kinematic test
SVM classifier (1)	87.61	86.40
SVM classifier (2)_SFM (30 epochs)	95.38	76.66
SVM classifier (2)_SFM (60 epochs)	95.90	73.86
SVM classifier (2)_SFM (90 epochs)	96.18	70.53
SVM classifier (2)_SFM (120 epochs)	96.23	67.86
SVM classifier (2)_ SFM (180 epochs)	95.76	65.92
SVM classifier (2)_ SFM (240 epochs)	95.63	64.68

**FIGURE 8:**
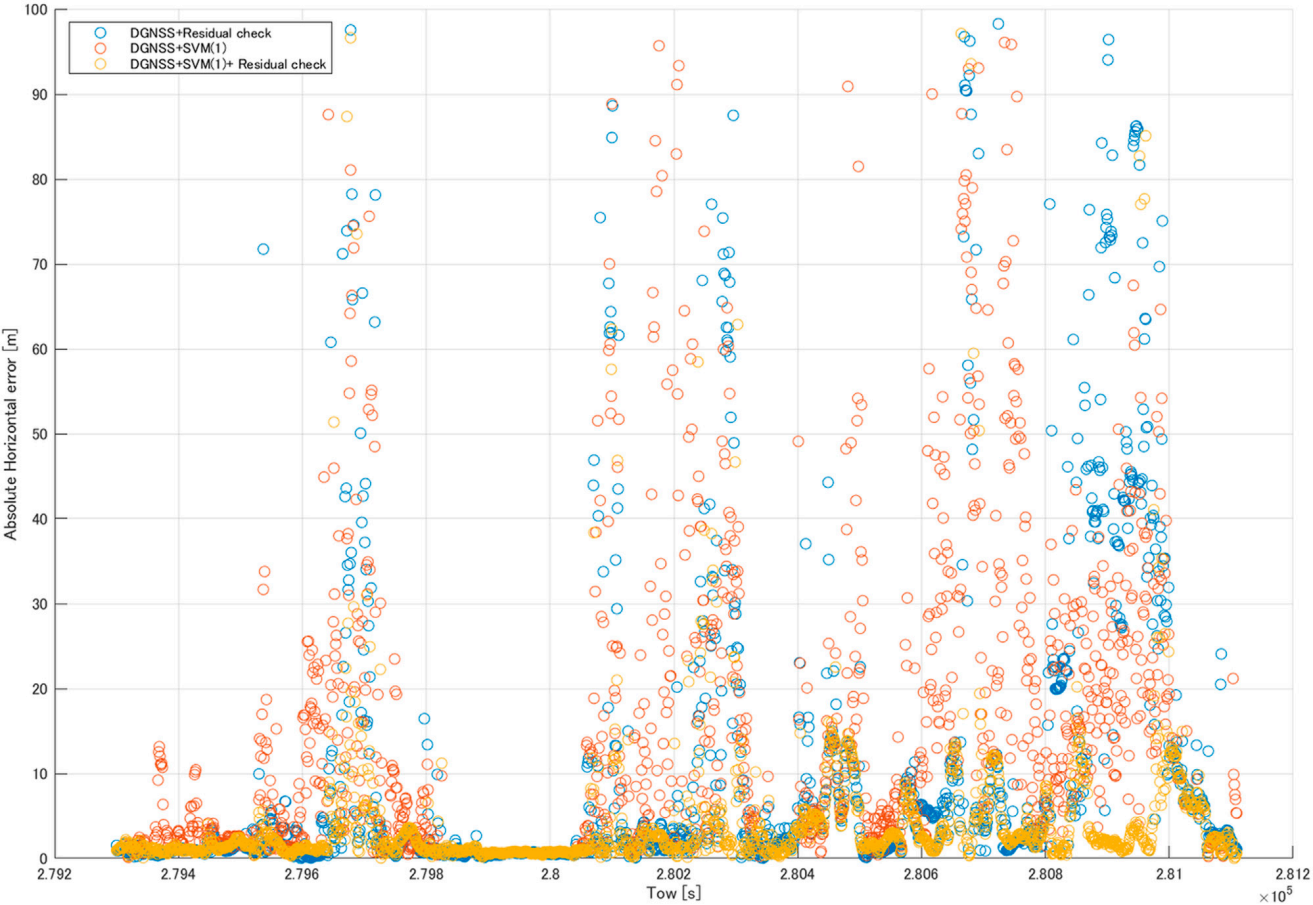
Comparison of the DGNSS positioning error in the kinematic test.

Table 7 shows a comparison of the horizontal positioning errors in the kinematic test, and Figure 8 shows the DGNSS positioning error with the DGNSS + residual check, DGNSS + SVM classifier (1), and DGNSS + SVM classifier (1) + residual check. Comparing the DGNSS and DGNSS + residual check, the pseudorange residual check was as effective as in the static test. As for DGNSS + SVM classifier (1), SVM classifier (1) was effective in positioning. However, it did not exceed the values of the DGNSS + residual check. For the combination of the DGNSS + SVM classifier (2) + residual check, all statistics were better as compared with the other methods.

**TABLE 7:** Comparison of the horizontal positioning error in the kinematic test.

Kinematic test (1,811 epochs)	DGNSS	DGNSS + residual check	DGNSS + SVM classifier (1)	DGNSS + SVM classifier (1) + residual check
Mean error	36.52 m	12.15 m	16.22 m	5.22 m
Maximum error	413.41 m	232.90 m	208.57 m	160.84 m
SD	43.20 m	21.95 m	23.61 m	11.97 m
Percentage (<3 m)	23.46%	50.80%	33.58%	64.38%
Percentage (<10 m)	35.37%	74.01%	57.10%	87.53%
Percentage (<30 m)	56.67%	86.17%	83.02%	97.72%

## 4 Discussion

We conducted two static tests and one kinematic test in this research study.

In the static test, the pseudorange residual check was effective, but the results of the DGNSS + residual check were not improved as compared with those of SVM classifier (2) based on Tables 4, 5. Additionally, the horizontal positioning results of SVM classifier (2) were better than those of SVM classifier (1), as indicated by the higher accuracy. Based on Table 3, the accuracy improvement of the training data gained by using the SFM was ∼8%, whereas for the test data, the improvement was >15%. Therefore, SVM classifier (2) was able to learn more effectively than SVM classifier (1) using the SFM under static conditions. A mean of 1.8 FN satellites at each epoch could not be eliminated in dataset (2), while a mean of 0.8 FN satellites at each epoch could not be eliminated in dataset (3). Thus, we could obtain more accurate positions with a combination of SVM classifier (2) and the pseudorange residual check (Tables 4, 5).

In the kinematic test, the pseudorange residual check was as effective as in the static test, while SFM did not contribute to the accuracy. The mean of the velocity in this experiment was 3.6 m/s, and if we were to calculate the SFM for 30 s, the positioning environment would change by approximately 100 m from start to end. Therefore, SFM does not indicate SNR fluctuations due to the NLOS signal. With a combination of SVM classifier (1) and the pseudorange residual check, we could obtain more accurate positions compared with the other methods (Table 7).

We proposed an SVM classifier and the combination of SVM classifier (1) and the pseudorange residual check to obtain a more accurate position. Notably, SFM was a useful feature under static conditions, whereas its use was problematic in kinematic tests. This is one of the novelties in static tests and a limitation in kinematic tests. The pseudorange residual check is useful in dense urban areas as evidenced by all tests in this study, whose effect is, however, limited when many NLOS signals are observed. On the other hand, the SVM classifier could not detect NLOS signals perfectly because its accuracy was approximately 90% in testing. Hence, as newly evidenced in this study, a combination of the SVM classifier and pseudorange residual check was the most effective method in dense urban areas.

Although the features used in this study are indicators of the characteristics of NLOS signals, it is difficult to determine from each feature whether or not a signal is an NLOS signal. It is also difficult to determine the NLOS thresholds for these features. This is where machine learning was introduced. The approach used here does not require other equipment and only requires an SVM classifier written in the Python code. In this study, we evaluated three datasets; however, all the data were obtained near Tokyo Station. To further assess the generality of the proposed method, it will be necessary to evaluate the classifications made using data from other locations. Moreover, we used data recorded for 27 min for training because there were few candidate locations at which NLOS signals could be observed with certainty, and it was difficult to park for long periods of time and obtain data. To further improve accuracy, we plan to find a place where we can obtain data for about 24 h and retrain the SVM classifier.

## Data Availability

The raw data supporting the conclusion of this article will be made available by the authors, without undue reservation.
